# HER-2 assessment in formalin-fixed paraffin-embedded breast cancer tissue by well-based reverse phase protein array

**DOI:** 10.1186/1559-0275-11-36

**Published:** 2014-10-01

**Authors:** Candice Perry, Catherine M Conway, Jeong Won Ha, Till Braunschweig, Jennifer Morris, Kris Ylaya, Hanbyoul Cho, Joon-Yong Chung, Stephen M Hewitt

**Affiliations:** Tissue Array Research Program, Laboratory of Pathology, National Cancer Institute, National Institutes of Health, Bethesda, MD 20892 USA; Institute of Pathology, RWTH Aachen University, Aachen, Germany; Department of Obstetrics and Gynecology, Gangnam Severance Hospital, Yonsei University College of Medicine, Seoul, 425-707 Korea; Antibody Characterization Laboratory, Advanced Technology Program, Leidos Biomedical Research, Inc, Frederick, MD USA

**Keywords:** Breast cancer, Formalin-fixed paraffin-embedded, Human epidermal growth factor receptor- 2, Immunohistochemistry, Reverse-phase protein array

## Abstract

**Background:**

The human epidermal growth factor receptor-2 (HER-2) expression level is a critical element for determining the prognosis and management of breast cancer. HER-2 targeted therapy in breast cancer depends on the reliable assessment of HER-2 expression status but current standard methods are lacking a rigorous quantitative assay. To address this challenge, we developed an assessment of HER-2 expression method by well-based reverse phase protein array (RPPA).

**Results:**

Well-based RPPA is based on a robust protein isolation methodology paired with a novel electrochemiluminescence detection system. HER-2 value of well-based RPPA significantly correlated with dot blotting results (R^2^ = 0.939). By well-based RPPA, we successfully detected HER-2 expression in 76 human breast formalin-fixed paraffin-embedded tissue samples. We observed 93.4% (71/76) concordance between well-based RPPA and current HER-2 immunohistochemical assessment guideline. When the cutoff level of HER-2 value was set to 0.689 (HER-2/GAPDH) on the basis of receiver-operating characteristic curve, the area under the curve was 0.975 (95% CI, 0.941-1.000). Sensitivity and specificity of well-based RPPA was 92.1% and 94.7%, respectively.

**Conclusions:**

HER-2 value by well-based RPPA was correlated with the current HER-2 status guideline, suggesting that this normalized HER-2 assessment may offer advantages over unnormalized current immunohistochemical assessment methods.

## Background

Human epidermal growth factor receptor-2 (HER-2), a proto-oncogene, encodes a tyrosine kinase receptor that functions to regulate cell growth, differentiation and survival [1, 2]. In malignant cells, overexpression of the HER-2 protein by *HER-2* gene amplification, leads to tumor development in 25-30% of invasive breast cancers and is associated with poor prognosis and shortened survival [3]. Furthermore, HER-2 is known to play an important role in patient selection of trastuzumab (Herceptin™®), a monoclonal antibody drug targeting the HER-2 protein [4–6]. In particular, 1 year treatment of trastuzumab after adjuvant therapy has shown to significantly improve disease-free survival for HER-2 positive breast cancer patients [6].

Determining the "optimized" method of detecting HER-2 overexpression remains controversial. The American Society of Clinical Oncology/College of the American Pathologists (ASCO/CAP) recommend using immunohistochemistry (IHC) and reflex testing of equivocal results by *in situ* hybridization (ISH) [7]. IHC is a simple and fast method that detects HER-2 protein expression on the cell surface by an antibody, and overexpression is based on HercepTest™ (DAKO, Glostrup, Denmark) or other assays and interpreted as scores of 0 and 1+ as negative and 2+ and 3+ as positive. IHC 3+ scores are more definitive; while IHC 2+ scores are considered equivocal and require further assessment by fluorescence *in situ* hybridization (FISH) [8]. While both methods are highly specific and reproducible when performed under standardized and validated conditions, IHC is semiquantative and staining interpretation is variable and subjective [9, 10]. Pre-analytic variables in the fixation and processing of breast specimens are well described to undermine HER-2 results. On the other hand, FISH assay requires expensive and technically difficult instrumentation [11]. In addition, the Food and Drug Administration (FDA) has approved chromogenic *in situ* hybridization (CISH, Spot-Light CISH) and ISH (Dual ISH) techniques for the evaluation of the amplification of the HER-2 gene, which do offer the precision of the FISH testing but on the morphometric evaluation of histological slides. However, this test is expensive and challenging to perform and interpret.

Reverse phase protein array (RPPA) is a sensitive and high throughput technology that allows the quantification of given makers in small amount of protein from biological specimens including formalin-fixed paraffin-embedded (FFPE) tissues [12]. This technique involves arraying protein samples on the substrate and then probing with the appropriate antibody, thus allowing various samples to be analyzed at the same time. However, current RPPA platforms require sophisticated printers and complicated study designs. To overcome these obstacles, we developed a well-based RPPA and showed the possibility as a powerful tool for proteomic profiling in clinical studies [13]. This platform does not require an arrayer and utilizes an electrochemiluminescence detection system.

In order to evaluate the benefits of well-based RPPA in HER-2 assessment, we extracted protein from 76 human breast cancer FFPE tissues and subsequently we performed a prospective study comparing HER-2 determination with IHC and well-based RPPA. We demonstrated that well-based RPPA effectively measured the negative and positive expression levels of HER-2 in breast FFPE tissue. This quantitative proteomic method is a powerful and reliable tool and can be used as an adjunct or as an alternative to IHC for optimal patient evaluation.

## Results

### Evaluation of HER-2 status by IHC completed FISH

**Figure 1:**
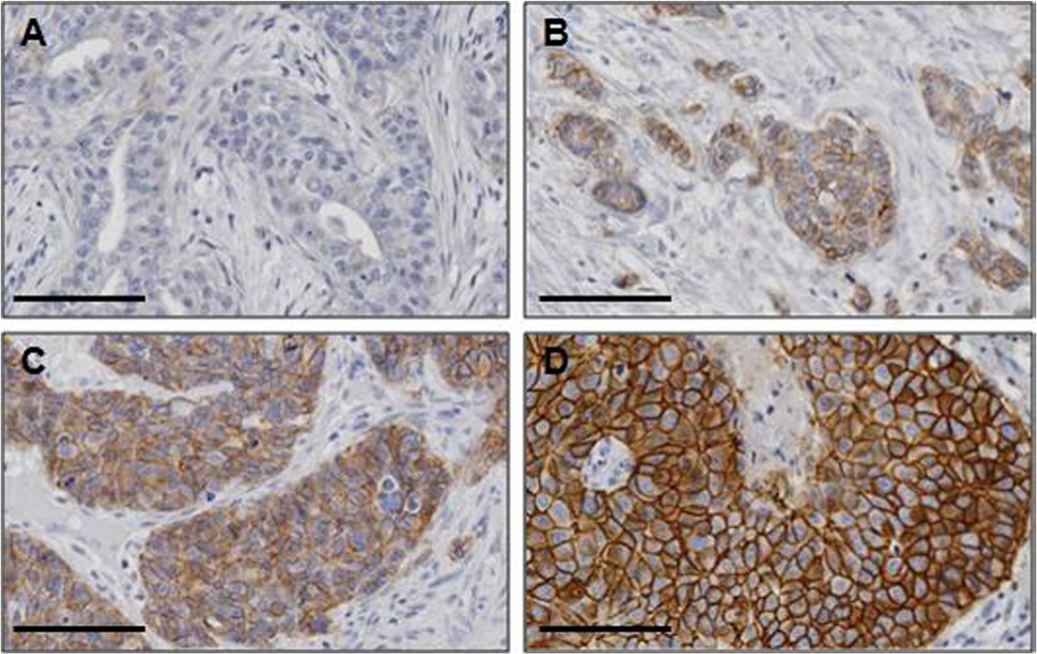
**Representative immunohistochemical staining images of HER-2.** The expression of HER-2 protein was detected by IHC. Representative HER-2 expression shown with 0 **(A)**, 1+ **(B)**, 2+ **(C)** and 3+ **(D)** subgroups. Scale bar: 100 μm.

Patients age ranged from 32 to 94 years (mean 49 years). Of the 76 patients, 54 cases were ductal carcinoma and 21 were lobular carcinoma. Representative images of HER-2 immunohistochemical staining are showed in Figure 1. In the semiquantitative analysis of HER-2 protein expression, the score was 0 in 10 cases (13.2%), 1+ in 22 (28.9%), 2+ in 15 (19.7%), and 3+ in 29 (38.2%), respectively. According to ASCO/CAP HER-2 scoring guideline, we performed FISH analysis for all IHC score 2+. Among the 15 equivocal cases, 9 were finally scored positive amplified by the overall HER-2 status, 6 cases were nonamplified.

### Quality validation of extracted protein from FFPE tissue

**Figure 2:**
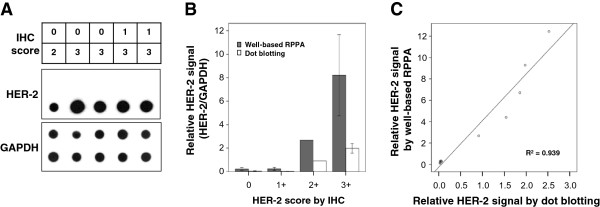
**Comparison of HER-2 expression levels by well-based RPPA and dot blotting. (A)** Dot blotting of HER-2 in breast cancer FFPE tissues. Protein extracted from 10 FFPE breast cancer tissue specimens and subsequently a total of 10 μg protein spotted on nictrocellulose membrane. The membrane was hybridized with HER-2 and GAPDH probes. HER-2 expression signals were only detected in 2+ and 3+ positive samples whereas GAPDH detected in all tested samples. Relative HER-2 expressional signals were calculated the ratio of HER-2/GAPDH. **(B)** Relative HER-2 expressional signals from four different IHC score subgroups were measured using well-based RPPA technology. Gray and white bars represent results of well-based RPPA and dot blotting, respectively. The bar graph shows the average ± SD of three replicated wells. **(C)** The signals from the well-based RPPA and dot blotting was strongly correlated for HER-2 expression status (R^2^ = 0.939).

To assess immunoreactive protein quality, we extracted protein from 10 archival human breast FFPE tissue specimens and subsequently performed dot blotting. Proteins extracted from archival human breast FFPE tissue specimens contained high amounts of immunoreactive proteins for HER-2 and glyceraldehyde 3-phosphate dehydrogenase (GAPDH) (Figure 2A). HER-2 expression was detected only in the HER-2 positive samples (2+ and 3+ score based on IHC) whereas GAPDH signal was shown in all tested samples. With the immunoreactive proteins, we analyzed whether the expressional signals from dot blotting analysis correlated with data from well-based RPPA. Relative HER-2 signals in series of breast FFPE tissue specimens were measured and the ratio of HER-2 to GAPDH was calculated. As shown in Figure 2B & C, the signals in well-based RPPA correlated with that of dot blotting analysis (R^2^ = 0.939), and had greater sensitivity.

### Evaluation of well-based RPPA methodology

**Figure 3:**
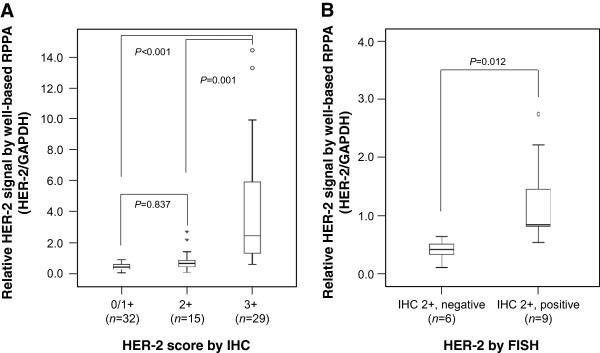
**Assessment of HER-2 expression status by well-based RPPA in human breast FFPE tissue specimens. (A)** Cases with 3+ score had significantly higher HER-2 expression than those with 0/1+ (*P* < 0.001) and 2+ score (*P* = 0.001). However, there is no significant difference between 0/1+ and 2+ subgroups (*P* = 0.837). **(B)** Tumor specimens with FISH positive showed significantly high HER-2 expressional values compare with that of FISH negative (mean of 1.233 versus 0.407, *P* = 0.012) in IHC 2+ subgroup. Relative HER-2 expressional signals were calculated the ratio of HER-2/GAPDH and the value expressed as box plot.

In order to evaluate diagnostic value of well-based RPPA, we investigated HER-2 status on all 76 cases and the result compared to the current guideline. By well-based RPPA, the HER-2 expression levels differed significantly between tested subgroup (0/1+ *vs.* 3+, *P* < 0.001; 2+ *vs.* 3+, *P* = 0.001). However, there was no significant difference between 0/1+ and 2+ subgroups (Figure 3A). Subsequently, we further analyzed equivocal group which are determined 2+ specimens by current immunochemical assessments. Notably, there is significant difference between FISH negative and FISH positive cases by well-based RPPA (Figure 3B, *P* = 0.012).

**Figure 4:**
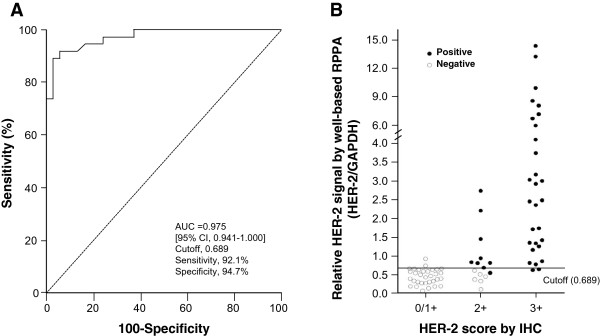
**HER-2 ROC curve and levels in breast cancer specimens. (A)** ROC curve for well-based RPPA assay results in distinguishing between HER-2 postive and negative cases. **(B)** Individual HER-2 expression levels showed in 0/1+ (*n* = 32), 2+ (*n* = 15) and 3+ (*n* = 29) subgroups. Positive (●) and negative (○) were categorized according to the current ASCO/CAP guideline. A value of cutoff was 0.0689.

Next we evaluated the diagnostic performance of well-based RPPA to determine HER-2 expression status. Figure 4A shows the ROC curve for the discrimination HER-2 expression status with positive *vs.* negative. The area under the curve (AUC) of well-based RPPA was found to be 0.975 (95% CI, 0.941 -1.000). A cutoff value of 0.689 (ratio of HER-2/GAPDH) had the highest accuracy (minimal false negative and false positive results) for HER-2 detection. Figure 4B shows the individual relative HER-2 value in the different IHC groups. Concordance was excellent in 0/1+ subgroup (93.8%) and 3+ group (93.1%). In addition, the well-based RPPA technology was showed great concordance (93.3%) with FISH in IHC 2+ subgroup whereas IHC showed lower agreement (60%, 9/15). Overall, the well-based RPPA showed great sensitivity and specificity, especially this methodology could be used substantial HER-2 expression status confirmation assay with advantages of excellent positive predictive value (94.6%) (Table 1).

**Table 1:** **Comparison of diagnostic value between IHC and well-based RPPA**

	Sensitivity (%)	Specificity (%)	PPV (%)	NPV (%)
**IHC**	100	84.2	86.4	100
**Well-based RPPA**	92.1	94.7	94.6	92.3

*Abbreviations*: IHC, immunohistochemistry; Well-based RPPA, well-based reverse phase protein array; PPV, positive predictive value; NPV, negative predictive value.

## Discussion

The accurate assessment of HER-2 in breast cancer is an imperative issue because HER-2 status is essential for identifying cancer patients who are appropriate for treatment with the anti-HER-2 humanized monoclonal antibody trastuzumab. Approximately 1.4% of patients who receive trastuzumab as a single agent [5, 14, 15] experience cardiotoxic side effects and this percentage increases when trastuzumab is combined with other chemotherapies (13% with paclitaxel and 27% with anthracyclines, respectively) [16], along with the cost of receiving therapy. Currently, the assessment of HER-2 status is performed predominantly by IHC with further validation being performed by FISH in the clinical laboratory.

IHC method measures the expression of the HER-2 protein on the surface of the tumor cells while FISH test measures the amplification of the *HER-2* gene present in the cells. The wide range of concordance rates between HER-2 protein expression by IHC and *HER-2* gene amplification by FISH has been well documented in several studies. In addition, IHC by comparison to FISH showed 21.8% false-positive rate and 8.9% false-negative rate at local laboratories [17]. The discordance between IHC and FISH assessment of HER-2 status in breast cancer reflects the wide variation in methodology, instrumentation, and experience of the laboratories performing the testing. Although the HercepTest is accurate and reproducible than laboratory developed test, the assessment of HER-2 by IHC is highly dependent upon both preanalytic factors and analytic factors [18, 19]. For these reasons, several methods for the evaluation of the HER-2 status have been introduced in addition to IHC, including Southern blot, slot blot, dot blot analysis, polymerase chain reaction, chromogenic *in situ* hybridization and immunoassay [20–26]. Many of these approaches focused on exploration at the gene expression level, and frequently require special instrumentation, technical expertise and can be time consuming. In addition, these methods did not employ a normalization tool which show very good correlation and low variance for data sets in quantitative proteomic approaches. Thus our methodology has an advantage that can evaluate HER-2 value without risk of low reliability and poor validity in the current immunohistochemical HER-2 assessment.

Notably, patients with high HER-2 expression at the 3+ by IHC showed approximately 35% (95% CI, 24.4-44.7%) response rate in randomized phase II study [5]. In this circumstance, there is a need for an accurate quantitative method to assess HER-2 protein expression using FFPE which can be performed in a clinical pathology setting. In this study, we propose that the use of this technology in HER-2 protein profiling can lead to fast and more reliable quantitative analysis. In order to successfully compare and justify implementation of this novel assay in accordance with approved clinical methods, we investigated the results from well-based RPPA in correlation with those of IHC and FISH data. Our study shows a high level of correlation between ASCO/CAP guideline and well-based RPPA (93.4%). Of particular interest were the 15 cases that were classified as 2+ when assessed using IHC. Six of the cases were considered to be positive by well-based RPPA and the remaining nine cases were negative. Only 2 out of the 15 would be considered borderline by well-based RPPA with a value of between 0.631 - 0.690 (HER-2/GAPDH). Therefore utilizing this protein array methodology as an adjunct to IHC would reduce the number of cases requiring further testing by FISH by 86.6%. Current tests that use inadequate post-surgical archival tissue specimens can compromise the accuracy of HER-2 status in a patient’s tumor such as fixation and preservation [27] and also observer variability due to tumor heterogeneity [28]. Although both aspects of variation can be addressed, the risks for false-positive and negative results are still prevalent.

The reverse phase protein array (RPPA) is a high-throughput technology which is suitable for monitoring changes in protein expression between disease and non-disease states [12]. In original RPPA technology, each lysate is arrayed in triplicates on nitrocellulose-coated slides in a dilution curve [29]. However, this approach is not compatible for large numbers of samples due to complexity of array printing and managing. In order to overcome these disadvantages, this platform was recently upgraded to a calibrated assay format like to ELISA [30]. Although this platform allows a single concentration printing instead of serial dilution of each lysate, this platform is also needs additional serial diluted reference panel which contain differing amounts of the target molecules, as well as high and low controls per each slide or matrix. Recently, Berg et al. reported a successful HER-2 assessment from 35 core biopsies and surgical specimens using RPPA, the authors tested only cancer cell rich tissue specimens (at least over 85% cancer cell) [31]. In addition, Wulfkuhle et al. demonstrated that HER-2 value by RPPA is concordant with FISH and/or IHC data [32]. Like RPPA, this well-based protein array methodology has the ability to quantify the levels of protein expression in a truly quantitative and continuous manner. Previously, we reported a successful protein extraction from FFPE tissue [13]. Our method has great protein extraction yield from relatively small amounts of material, with relatively low concentration of detergent regardless of the deparaffinization step. Prior to use of an antibody in well-based RPPA, its specificity was validated by dot blot (Figure 2). It should be noted that the well-based RPPA used 25 fold less protein than the dot blot. Subsequently, HER-2 assay by well-based RPPA sit in the linear dynamic range (data not shown). Finally, the HER-2 signal was normalized to GAPDH protein, similar to western blotting. Our assay showed a high specificity of 94.7% than classical IHC analysis of 84.2%. While the sensitivity was lower than IHC, the results may reflect potential false positives commonly seen in IHC.

Basically, our well-based RPPA does not require the use of a printer arrayer, nor does it use scoring a dilution curve. Furthermore, one substantial advantage is the capacity to measure multiple proteins and develop a normalized metric based on expression of a largely invariant protein. For the best results, the researcher should be checking the dynamic range against the target molecule, using antibody which have great specificity and sensitivity prior to the real experiment. As most of proteomic assay, this methodology is also mainly depending on the antibody quality.

HER-2 status can be assessed on both surgical specimens and core biopsies by IHC and FISH. These both methodologies can be influenced by pre-analytical factors including warm ischemia, fixation time and tissue processing conditions. We suppose the assay quality of well-based RPPA is also partially linked with pre-analytical variables. However, the pre-analytical variables are less impactful on the result of this new methodology because the new array is very sensitive and specific assay, as well normalizing for cellular content and pre-analytic factors that impact tissue quality.

The protein quality of FFPE tissue shows a "smear" on SDS-PAGE and the extent of smearing represents the specimen quality. For example, greater smearing indicates different degradation levels for each tissue samples. This means the total protein alone is not an appropriate normalization tool in the FFPE tissue samples. In a previous study, we showed that GAPDH protein signal is a good indicator as an internal control to measure the protein quality from each tissue sample [33]. Thus, we calculated the HER-2 signal based on double normalization with total amount of protein and the GAPDH signal. This approach has an advantage that can evaluate HER-2 value without risk of low reliability.

## Conclusions

In summary, we have demonstrated the use and applicability of a well-based RPPA for HER-2 detection and quantification with normalization. The comparative data analysis between well-based RPPA and FISH were highly correlated in the equivocal subgroup indicating well-based RPPA would be more useful in predicting anti-HER2 therapy in patient clinical management. Additional evaluation is required, optimally with breast cancer cases that received Herceptin, to determine if this approach can outperform the current IHC based method, which has a weak positive predictive value in identification of patients who will respond to Herceptin treatment.

## Materials and methods

### Tissue specimens

A total of 76 formalin-fixed, paraffin-embedded (FFPE) breast cancer specimens were obtained from the collection of the Tissue Array Research Program (TARP), Bethesda, MD and anonymized breast cancer tissue samples of the laboratory of Pathology at RWTH Aachen University, Aachen, Germany. Material was obtained with appropriate human protection approvals from the institutional review board of the RWTH Aachen university hospital and Office of Human Subjects Research at the NIH.

### Immunohistochemistry

Breast cancer specimens were assessed for HER-2 status using a DAKO autoimmunostainer (DAKO, Carpinteria, CA). Briefly, whole sections (5 μm) were deparaffinized with xylene and rehydrated through graded alcohols to distilled water. Endogenous peroxidase activity was blocked with 0.3% hydrogen peroxide for 10 minutes, and antigens were retrieved for 40 minutes in 10 mmol/L citrate buffer, pH 6.0, with steamer. The whole section slides were incubated with the rabbit anti-human HER-2 polyclonal antibody A0485 (1:500 dilution; DAKO) for 30 minutes at 25°C. Slides were then incubated with a DAKO Envision^+^ for 30 minutes at 25°C, subsequently reacted with 3,3′-diaminobenzidine as a chromogen substrate for 3 minutes, counterstained using a Modified Harris hematoxylin (Thermo Scientific, Rockford, IL) and coverslipped after dehydration.

### IHC interpretation and scoring

All IHC cases were digitized utilizing at 20× magnification using an Aperio CS scanner (Vista, CA) and the images were reviewed in SlidePath software (Leica, Buffalo Grove, IL). The reviewers adhered to the ASCO/CAP guidelines for HER-2 interpretation of invasive breast cancer. Therefore, all cases were classified as one of the following; 0, no staining observed, or membrane staining observed in < 10% of tumor cells; 1+, a faint/barely perceptible membrane staining is detected in ≥ 10% of tumor cells, the cells exhibit incomplete membrane staining; 2+, a weak to moderate complete membrane stain is observed in ≥ 10% of tumor cells; 3+, a strong complete membrane staining is observed in ≥ 10% of tumor cells.

### Fluorescence in situ hybridization

Of all the cases classified by IHC as HER-2/neu 2+, FISH analysis was performed. For fluorescence *in situ* hybridization (FISH), the PathVysion kit by Abbott was used (02 J01-035, Abbott Laboratories, Abbott Park, IL). As used for IHC, 5 μm sections of paraffin blocks were processed following manufacturers guidelines (deparaffination, rehydration, pretreatment, enzyme treatment, fixation, hybridization of probes, DAPI counterstain, coverslipping). Two probes are included in the kit, one probe against the HER-2/neu locus, 17q11.2-q12 LSI, fluorescent to SpecOrange, the second probe against centromer of chromosome 17 (CEP17), fluorescent to SpecGreen. In 20 cells, both signals were counted of each nucleus followed by building a ratio (HER-2/neu to CEP17) of the sums of signals. Below a ratio of 2, HER-2/neu was considered not amplified, a ratio of 2 and up was considered amplified. The analysis was performed on an inverse microscope by Zeiss, Axiovert 100 using Zeiss fluorescent filter sets for both probes (Zeiss, Thornwood, NY).

### Protein extraction

Protein extraction from archival FFPE tissue was performed as previously described [13]. Briefly, two 10 μm sections of each specimen were lysed with an extraction buffer [1x high pH Antigen retrieval buffer (DAKO), 1% NaN3, 1% SDS, 10% glycerol, and protease inhibitor (1 tablet/25 ml, Roche, Basel, Switzerland)]. Afterwards, incubation for 15 mins at 115°C within a pressure cooker followed by microcentrifugation at 13000 rpm for 30 mins at 4°C. Protein was stored -20°C until further use. Protein concentrations were determined using BCA protein assay (Pierce Biotechnology, Rockford, IL) and used according to the manufacturer’s instructions.

### Dot Blot

The protein lysates (10 μg) extracted from human breast FFPE tissue sections was dotted to 0.2 μm nitrocellulose membrane using Bio-Dot Microfiltration Apparatus (Bio-Rad, Hercules, CA). The membranes were blocked in 5% nonfat dry milk in TTBS (50 mM Tris, 150 mM NaCl, 0.05% Tween 20, pH 7.5) for 1 hour at room temperature (RT), washed, and then incubated overnight in 5% BSA in TTBS at 4°C containing antibodies; HER-2 polyclonal antibody (DAKO, 1:2000) and anti-GAPDH (Calbiochem, Gibbstown, NJ, 1:1000). Subsequently, the membranes were washed and incubated with horseradish peroxidase labeled anti-rabbit and anti-mouse secondary antibody for 90 minutes and enhanced using a SuperSignal Chemiluminescence kit (Pierce Biotechnology) and detected on Kodak Biomax MR X-ray film (Kodak).

### Well-based reverse phase protein array

In order to quantitate HER-2 expression values, we performed well-based reverse phase protein array (RPPA) as previously reported [13]. Briefly, extracted proteins (400 ng) were applied onto 96-well Multi-Spot™ plates (Meso Scale Discovery, Gaithersburg, MD, MA2400 96 HB Plate), the plate was allowed to dry at RT, and if needed, further incubated at 37°C for 30 min. The antigen-coated plates were pre-incubated with 5% BSA in PBST for 60 min at RT before primary antibody incubation. Anti-HER-2 (DAKO) and anti-GAPDH (Calbiochem) were diluted 1:1000 and 1:5000 with 5% BSA in PBST, followed overnight incubation at 4°C. After washing with PBST, the plates were incubated for 90 min with goat anti-rabbit or mouse SULFO-TAG™ antibodies (Meso Scale Discovery) at a dilution of 1:2000. The plates were washed three times with PBST. MSD-T read buffer was added to the plates and signals were detected using Sector Imager 2400 reader (Meso Scale Discovery). BSA coated wells were included on each plate as a control of non-specific binding. HER-2 expression signal was normalized with the value of GAPDH.

### Statistical analysis

Statistical analysis was performed using IBM SPSS Statistics 19.0 (SPSS Inc., Chicago, IL). Comparison of means among the three groups (0/1+, 2+ and 3+) was performed using one-way ANOVA post hoc test. The accuracy of well-based RPPA for determination of HER-2 positive or negative breast cancer tissue samples was evaluated using receiver-operating characteristic (ROC) curve. Sensitivity, specificity, positive predictive values (PPVs), negative predictive values (NPVs) and their corresponding confidence intervals (CIs) were calculated. A *P*-value < 0.05 was considered as statistically significant.

## Consent

Written informed consent was obtained from the patient for the publication of this report and any accompanying images.

## Abbreviations

FDA: Food and Drug Administration
FISH: Fluorescence *in situ* hybridization
GAPDH: Glyceraldehyde 3-phosphate dehydrogenase
HER-2: Human epidermal growth factor receptor-2
IHC: Immunohistochemistry
ISH: *In situ* hybridization
RPPA: Reverse phase protein array
PPV: Positive predictive value
NPV: Negative predictive value.
